# Contrast variation method applied to structural evaluation of catalysts by X-ray small-angle scattering

**DOI:** 10.1038/s41598-024-52671-7

**Published:** 2024-01-27

**Authors:** Albert Mufundirwa, Yoshiharu Sakurai, Masazumi Arao, Masashi Matsumoto, Hideto Imai, Hiroyuki Iwamoto

**Affiliations:** 1 Research Project Division, Japan Synchrotron Radiation Research Institute, SPring-8, Sayo-Cho, Sayo-Gun, Hyogo 679-5198 Japan; 2 Fuel Cell Cutting-Edge Research Center Technology Research Association, 3147, Shimomukouyama-Cho, Kofu, Yamanashi 400-1507 Japan

**Keywords:** Energy science and technology, Nanoscience and technology

## Abstract

In the process of developing carbon-supported metal catalysts, determining the catalyst particle-size distribution is an essential step, because this parameter is directly related to the catalytic activities. The particle-size distribution is most effectively determined by small-angle X-ray scattering (SAXS). When metal catalysts are supported by high-performance mesoporous carbon materials, however, their mesopores may lead to erroneous particle-size estimation if the sizes of the catalysts and mesopores are comparable. Here we propose a novel approach to particle-size determination by introducing contrast variation-SAXS (CV-SAXS). In CV-SAXS, a multi-component sample is immersed in an inert solvent with a density equal to that of one of the components, thereby rendering that particular component invisible to X-rays. We used a mixture of tetrabromoethane and dimethyl sulfoxide as a contrast-matching solvent for carbon. As a test sample, we prepared a mixture of a small amount of platinum (Pt) catalyst and a bulk of mesoporous carbon, and subjected it to SAXS measurement in the absence and presence of the solvent. In the absence of the solvent, the estimated Pt particle size was affected by the mesopores, but in the presence of the solvent, the Pt particle size was correctly estimated in spite of the low Pt content. The results demonstrate that the CV-SAXS technique is useful for correctly determining the particle-size distribution for low-Pt-content catalysts, for which demands are increasing to reduce the use of expensive Pt.

## Introduction

Small-angle X-ray scattering (SAXS) is a useful method for characterizing the structure of catalyst particles for fuel cells and other applications^1,2^. In these applications, the nanometer-sized catalyst particles, typically made of platinum (hereafter Pt), are supported on larger particles made of lighter elements, typically carbon^3–7^. Determining the particle-size distribution of the catalysts is important, because it allows for the estimation of the catalytic surface area, which is directly related to the catalytic activity of the whole system. The growth of catalyst particles during fuel cell operation is a major source of catalyst degradation.

Particle sizes can be determined by several techniques, including SAXS, wider-angle X-ray diffraction (XRD), X-ray absorption fine structure (XAFS) and transmission electron microscopy (TEM)^8,9^. Among these techniques, only SAXS and TEM can determine the distribution of particle sizes. SAXS is superior to TEM in terms of counting statistics, because in SAXS measurements, a far greater number of catalyst particles exist in the X-ray beams than in the observed areas of TEM.

A drawback of SAXS measurements is that they cannot distinguish particles of heterogeneous nature if their sizes are similar. This limitation arises because X-ray scattering is sensitive only to the spatial distribution of electrons that are common to all elements. In the case of carbon-supported Pt catalysts, the contribution of carbon can usually be ignored, because the number of electrons per atom (atomic number) is far greater for Pt than for carbon (78 vs. 6, the scattering intensity is proportional to the square of the atomic number). However, if only a small number of Pt particles exist on carbon, the contribution of carbon cannot be ignored. This situation could result in erroneous estimation of particle size distributions, especially when the carbon has mesopores with sizes similar to those of Pt catalysts.

One way to overcome this problem would be through the use of anomalous SAXS (ASAXS, e.g.^10–12^). This method utilizes the phenomenon known as anomalous X-ray scattering, in which the scattering intensity changes sharply with X-ray energies at around the absorption edge of a specific element. By employing this technique, structural information of a single element (here, Pt) can be extracted from a multi-element system. However, the energy-dependent change of scattering intensity is usually very small (equivalent to only a few electrons per atom), and it can introduce errors unless very careful and accurate measurements are carried out.

Here we propose an alternative method to address this issue, namely the use of contrast-variation SAXS (CV-SAXS). In this method, the sample is immersed with a solvent. When the electron density of the solvent matches that of one of the components in the sample (here, carbon), there will be no contrast between the solvent and the component, causing the component to become invisible to X-rays. In the Pt-carbon system, only Pt should be visible to X-rays at the matching density, allowing one to determine its size distribution without being influenced by carbon microstructure. The advantage of CV-SAXS is that, unlike ASAXS, the requirements for measurements are not stringent, and the X-ray energy is not restricted to the range around the absorption edge of the element of interest.

The contrast-variation method is widely used in small-angle neutron scattering (SANS)^13–17^. For example, hydrogen and deuterium have very different scattering cross sections for SANS, and by replacing all the hydrogen atoms in a component with deuterium, it becomes possible to render that component invisible to neutrons. Although less frequently, the contrast-variation method is also utilized in SAXS, especially in protein solution scattering experiments^13,15,18,19^. In this scenario, distinct protein species have slightly different electron densities, and the electron density of the solvent can be adjusted to match that of one protein species by dissolving different amounts of sucrose or other solutes.

We needed to start with finding a solvent system suitable for metal catalyst-carbon systems. We found that a solvent density can be adjusted to be around that of carbon, when a high-density solvent 1,1,2,2-tetrabromoethane (TBE) is mixed with lower-density dimethyl sulfoxide (DMSO) in various ratios. First, we determined the matching concentration of TBE for carbon. Then, we applied the CV-SAXS technique to a commercially available Pt catalyst supported on solid carbon and a mesoporous carbon sample with mesopores whose size was comparable to that of Pt catalysts. Mesoporous carbon has attracted attention as support for catalysts, due to its superior properties as compared with conventional solid carbon, such as increased surface-to-volume ratio^20^. Therefore, it is anticipated that the demands for a method to accurately characterize catalysts supported by mesoporous carbon will increase.

Here we show that the CV-SAXS technique can effectively suppress the SAXS features originating from carbon mesopores, and this technique can be readily applied to extract the structural information of catalyst particles alone.

## Materials and methods

The Vulcan XC72-supported Pt catalyst, TEC10V30E, was purchased from Tanaka Precious Metals, Japan. This was used without any pre-treatment (as-made). The carbon samples, Vulcan XC72 and CNovel MH00, were obtained from Cabot Corporation (USA) and Toyo Tanso (Japan), respectively. 1,1,2,2-tetrabrmoethane (TBE) and dimethyl sulfoxide (DMSO) were obtained from Tokyo Chemical Industry (Japan) and Merck (Germany), respectively. The concentration of TBE in the solvent is expressed in volume/volume (the volume change upon mixing is negligible and can be practically ignored).

**Figure 1:**
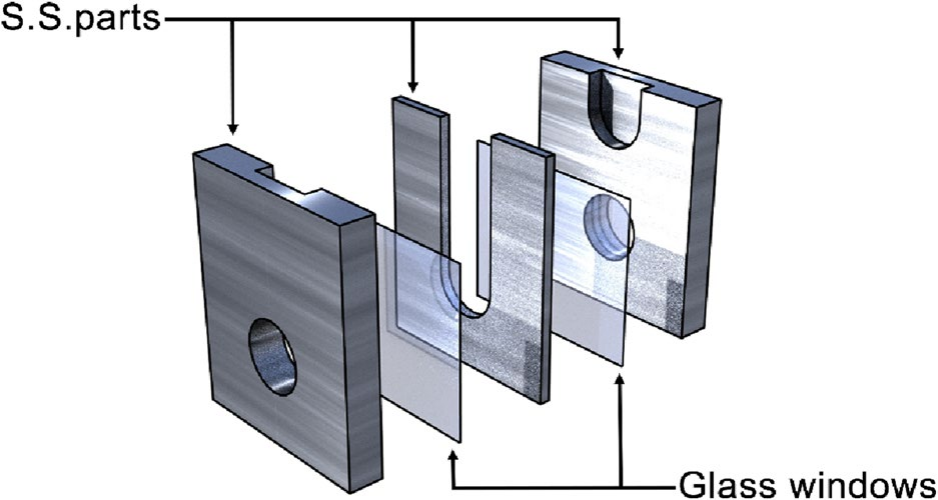
Structure of microcell. Three stainless-steel (S.S.) parts and two glass windows were glued together. The dimensions of a finished cell were 6 mm (W) × 7 mm (H) × 2.36 mm (D).

The SAXS measurements were carried out at the BL40B2 beamline in SPring-8, Hyogo, Japan. This beamline is dedicated to SAXS, and has a small-angle resolution of *q* ~ 0.007 nm^−1^. The samples, either in the form of dry powder or suspensions in solvents, were filled in a microcell (Fig. 1) with two glass (BK-7) windows (thickness, 30 µm), and the space between the windows was 300 µm. This spacing was chosen after considering the X-ray absorption by TBE that contains bromide. The diameter of the window (2 mm) was greater than the beam size (0.6 × 0.35 mm, horizontal × vertical). Its minimal volume was approximately 1 µL.

A fixed amount of sample was thoroughly dispersed in the TBE/DMSO mixture (28.6 mg/mL) in a polypropylene microtube immediately before X-ray recording. A 5 µL aliquot was pipetted to the cell. Sometimes it was necessary to spin down the suspension to the bottom of the microcell by using a hand-driven centrifuge. The uniformity of the suspension was inspected by observed through the window. If any non-uniformities were detected, the suspension was further stirred with a very thin stainless-steel wire.

The X-ray scattering was recorded with a 2-dimensional X-ray detector, Pilatus 2 M (Dectris, Switzerland). The sample-to-detector distance was 1.2 m and the X-ray energy was 11.504 keV. This energy is below the absorption edges of Pt LIII (11.564 keV) and Br K (13.474 keV) so that the absorption from Pt and Br and their fluorescence were kept at low levels. A vacuum path was placed between the sample and the detector. The X-ray flux was 10^11^ photons/s. The total exposure time for each sample was 100 s (10 s × 10 exposures). The 2-D data were later reduced to 1-D, and the blank data (cell only or cell with solvent only) was subtracted after correction of absorption, to obtain the actual scattering intensities from the sample. For this purpose, two ionization chambers were placed just upstream and downstream of the sample to measure X-ray fluxes. The following equation was used for correction:

$$I\left( {{\text{SL}}} \right) = I\left( {{\text{SL}} + {\text{SV}} + {\text{C}}} \right) - I\left( {{\text{SV}} + {\text{C}}} \right) \cdot \left( {T\left( {{\text{SL}} + {\text{SV}} + {\text{C}}} \right)/T\left( {{\text{SV}} + {\text{C}}} \right)} \right),$$

where *I* is intensity and *T* is transmittance. SL, SV and C stand for solute, solvent and cell, respectively. Transmittance is defined as *I*(anything)/*I*(air).

## Results

### Determination of matching concentration of TBE for carbon samples

We first determined the matching concentration of 1,1,2,2-tetrabromoethane (TBE) for carbon samples. The intensity of X-ray scattering is proportional to the square of electron density (number of electrons per unit volume). When the electron density of the solvent matches that of the carbon sample, there is no contrast, and consequently, the carbon becomes invisible to X-rays. As carbon samples, we used Vulcan XC72 (solid carbon) and CNovel MH (mesoporous carbon), both commonly used for supporting metal catalyst particles. The electron densities can be calculated using the chemical formulae of the substances and their specific gravities. The specific gravity of graphite is reported to be 2.09–2.23^21^. By using a value of 2.2 as the specific gravity and the atomic number of carbon (6), the electron density of graphite is calculated to be 1100 mol/L (1100 × Avogadro’s number of electrons in a L). Likewise, the electron densities of TBE and DMSO are calculated to be 1322 and 592 mol/L, respectively, by using specific gravities described in the manufacturer’s data sheets. The electron density of TBE exceeds that of graphite, so that it is expected that one can prepare a solvent with a matching density by mixing it with DMSO in an appropriate ratio.

**Figure 2:**
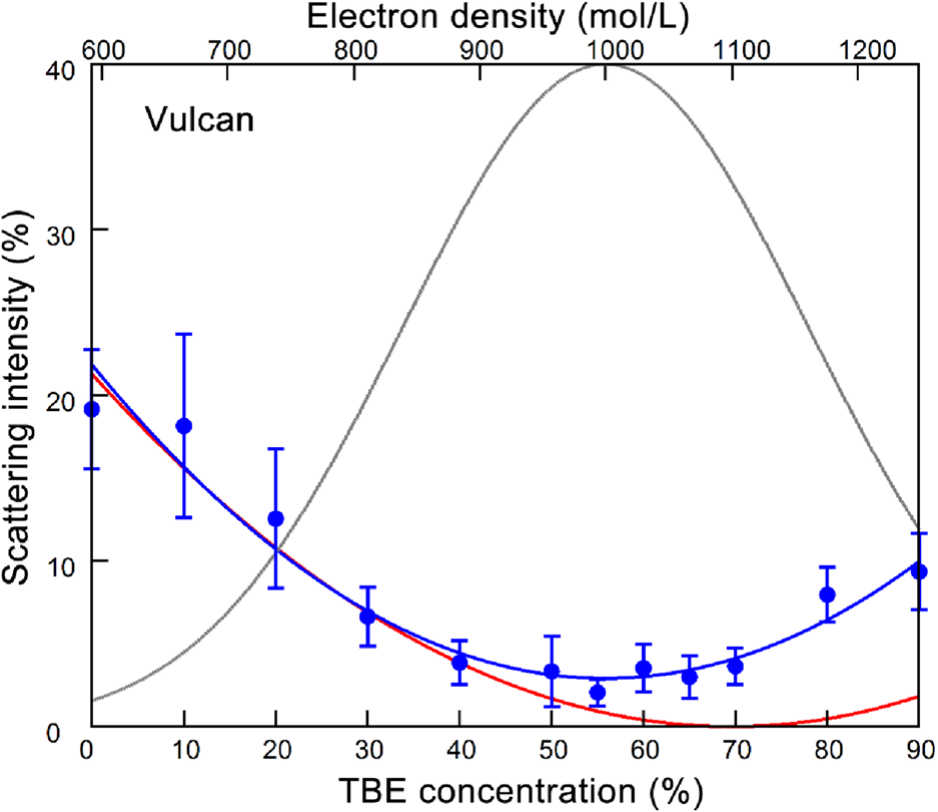
Dependence of the intensity of X-ray scattering from Vulcan on the concentration of tetrabromoethane (TBE) in the solvent. The intensity is relative to that in air. Blue circles, observed values of scattering intensities (mean ± S.D., n = 8–11). These values are normalized so that the value at 0% TBE coincides with the theoretical value (red curve). Red curve, theoretical values of scattering intensity when the electron density of carbon is 1100 mol/L. Blue curve, best-fit scattering curve when the electron density of the carbon has a Gaussian distribution (gray curve; 1000 ± 160 mol/L.).

We prepared a series of TBE/DMSO mixtures with TBE concentrations ranging from 0 to 90%, dispersed carbon samples (28.6 mg carbon/mL), and recorded the intensity of X-ray scattering. Figure 2 shows the dependence of scattering intensities from Vulcan on TBE concentration. The intensity was integrated over a *q* range between 0.1 and 6 nm^−1^. This range is far above the small-angle resolution of the beamline (*q* ~ 0.007 nm^−1^) and practically free of parasitic scattering. The conclusion presented here is unchanged when the integration range is limited to a higher-*q* region. By using the specific gravity value of 2.2, the expected matching concentration is approximately 70%. The scattering intensities decreased with increasing TBE concentration, reaching their lowest point at 50–60%. The scattering intensity did not reach zero, meaning that the true matching point did not exist. This situation can arise if the electron density of the carbon sample is not uniform. Such a material can be regarded as a mixture of particles with varying matching concentrations.

The blue curve in Fig. 2 is the best-fit theoretical curve, assuming that the electron density of the carbon sample follows a Gaussian distribution (1100 ± 160 mol/L, mean ± S.D.). This fit demonstrates a substantially wide distribution of electron density within the carbon sample. The scattering curve from CNovel shows similar tendencies (Supplemental Material).

Although the true matching point is not reached, the scattering from the carbon samples at the bottom of the scattering curve is only a few percent of that in the air. Because of this, these concentrations are practically considered as matching concentrations. Hereafter, we will use a 50% TBE/DMSO mixture as a solvent with a practically matching concentration.

### X-ray scattering curves for Vulcan and Vulcan-supported catalysts

**Figure 3:**
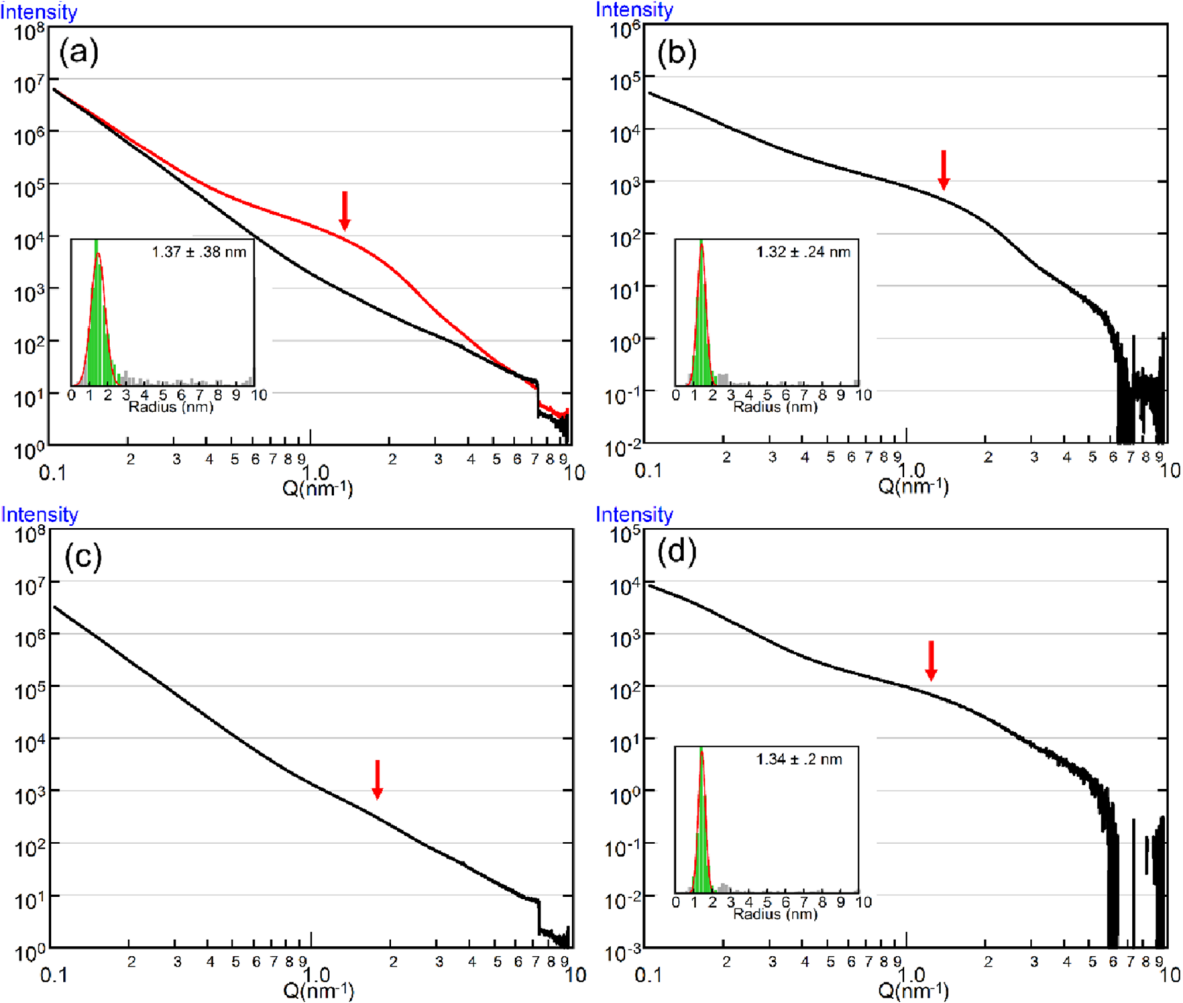
Scattering curves from Vulcan and Vulcan-supported catalysts. (**a**) Scattering curves from Vulcan (black) and TEC10V30E (red) in the absence of solvent. The curve from TEC10V30E exhibits a prominent shoulder-like feature at around *q* = 1.0 nm^−1^ (arrow), originating from Pt particles. The two curves are normalized to coincide at the low-*q* end. The inset shows the distribution of Pt particle size, obtained by McSAS. The red curve represents the fit to a Gaussian function. (**b**) Scattering curve from TEC10V30E dispersed in 50% TBE after subtracting the scattering from the solvent alone. The shoulder-like feature remains as prominent as in (**a**), and the Pt particle size as determined by McSAS is not different from that in the absence of the solvent. (**c**) Scattering from a mixture of TEC10V30E and Vulcan (TEC = 6% by weight) in the absence of solvent. The shoulder-like feature is barely recognized, and cannot be reliably analyzed by McSAS. (**d**) Scattering from a mixture of TEC10V30E and Vulcan (TEC = 5% by weight) dispersed in 50% TBE. The shoulder-like feature is clearly observed, and is analyzable by McSAS.

Figure 3a shows the scattering curves for Vulcan and a commercially available Vulcan-supported Pt catalyst, TEC10V30E (catalyst ratio, 30% wt.), filled in glass capillaries without a solvent. The scattering intensity for Vulcan decreases monotonically with increasing scattering angle (*q*-value), and does not show any features. On the other hand, the scattering curve for TEC10V30E has a prominent shoulder-like feature at around *q* = 1 nm^−1^. This feature represents the Guinier-region for the Pt catalyst particles, and by analyzing this part of the curve, one can determine their size distributions. Here we used the freely downloadable software McSAS^22,23^ to determine the particle size distribution, which was 2.74 ± 0.76 nm in this case (mean diameter ± standard deviation after fitting the distribution with a Gaussian function). McSAS is a software package that employs Monte-Carlo regression to fit the model data to observed scattering curves, and obtains particle size distributions. McSAS was validated by model data and the obtained particle size distribution shows good agreement with that obtained from TEM data (see Supplemental Material).

Next, we recorded the scattering curve for TEC10V30E dispersed in 50% TBE/DMSO (Fig. 3b). The scattering from the carbon was suppressed while that from Pt was not, and as a result, the shoulder-like feature remained prominent.

Finally, we dispersed a mixture of TEC10V30E and Vulcan to simulate a sample with a low catalyst ratio. The content of TEC10V30E was 5–6% by weight, so that the final catalyst ratio was 1.5–2%.When this sample is measured without a solvent, the feature of the Pt particles is obscured by the bulk of free Vulcan particles, making an accurate estimation of particle size distribution difficult (Fig. 3c).

In 50% TBE/DMSO, however, the scattering from Vulcan was suppressed, and the feature of the Pt particles was once again clearly observed (Fig. 3d). We were able to determine the particle size distribution, which was 2.68 ± 0.4 nm and was not different from the value determined for TEC10V30E alone.

### X-ray scattering from CNovel and its mixture with TEC10V30E

Finally, we proceeded to use mesoporous CNovel carbon particles instead of Vulcan. Several varieties of CNovel are commercially available, but we chose MH00 because its mesopore diameter (~ 4 nm) is comparable to the size of Pt nanoparticles.

**Figure 4:**
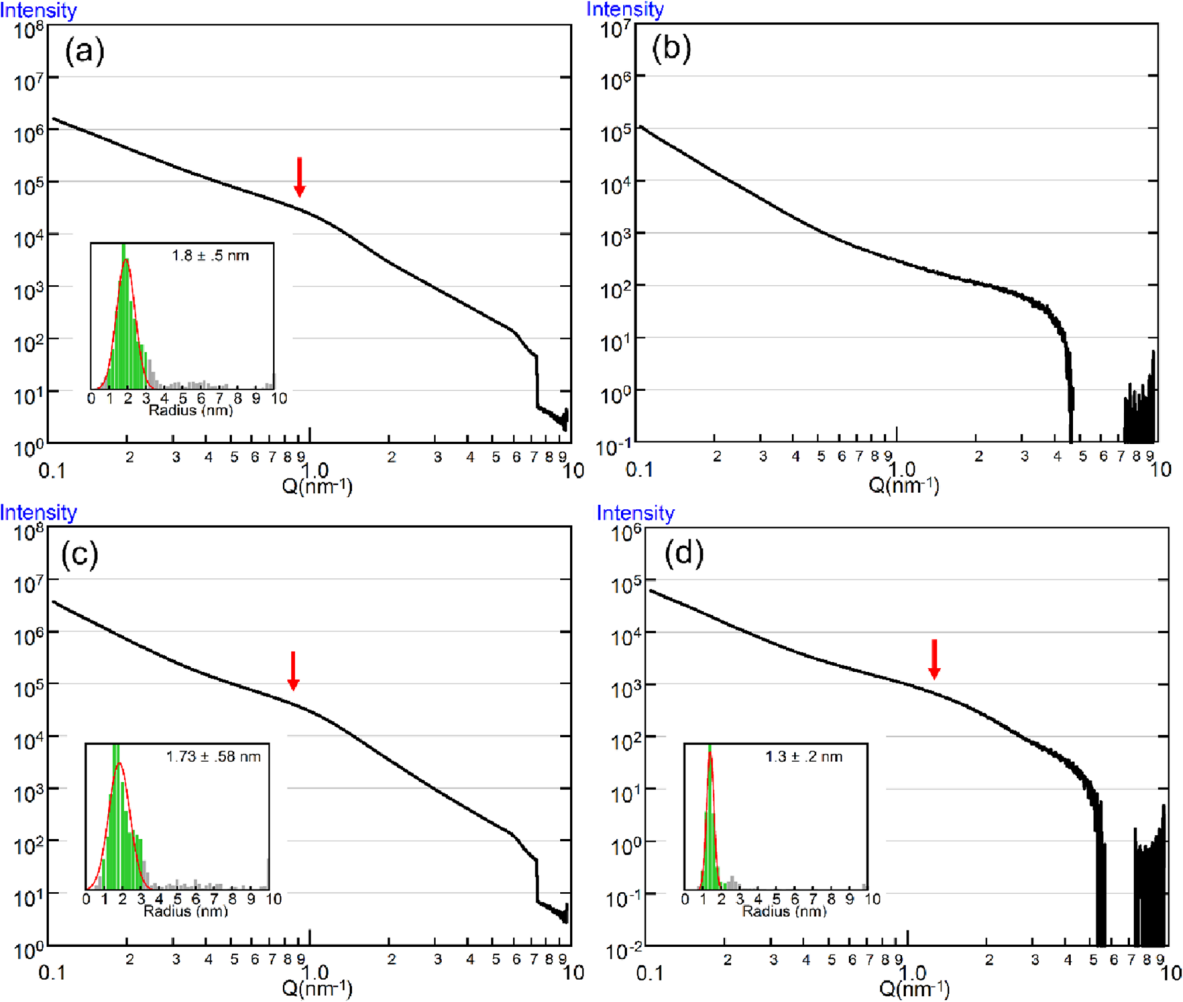
Scattering curves from CNovel and its mixture with a low content of TEC10V30E. (**a**) Scattering curve from CNovel MH00 in the absence of solvent. It exhibits a shoulder-like feature (arrow) at *q*-values slightly higher than those for TEC10V30E (see Fig. 3a), and it is analyzable by McSAS (inset). (**b**) Scattering curve from CNovel MH00dispersed in 50% TBE. Note that the shoulder-like feature is no longer observable. (**c**) Scattering curve from a mixture of TEC10V30E and CNovel MH00 (TEC = 15% by weight) in the absence of solvent. The shoulder-like feature is similar to that of CNovel alone (**a**). (**d**) Scattering curve from a mixture of TEC10V30E and CNovel MH00 (TEC = 15% by weight) dispersed in 50% TBE. The shoulder-like feature remains recognizable, but it is positioned at slightly higher *q*-values than in (**a**) and (**c**).

Figure 4a shows the scattering profile of CNovel particles in the absence of a solvent. Although the mesopores have negative contrast against the carbon body, their effects on X-ray scattering are the same as those with positive contrast. As a result, a Guinier-like feature is observed in the scattering curve and it is almost indistinguishable from that originating from catalyst particles. The size distribution of the mesopores can also be analyzable by McSAS, and it was determined to be 3.6 ± 1.0 nm, which is slightly larger than the Pt particles in TEC10V30E.

When CNovel particles were dispersed in 50% TBE, the Guinier-like feature was no longer observable (Fig. 4b). This observation confirms that the feature originates from the nano-structure of carbon.

Next, we mixed CNovel and TEC10V30E to simulate CNovel-supported Pt catalysts. The content of TEC10V30E was again ~ 15% by weight so that the catalyst rate was ~ 4.5%. In the absence of a solvent, the scattering curve (Fig. 4c) is very similar to that from CNovel alone (Fig. 4a), and a Guinier-like feature is observed at the same *q*-values. The particle size distribution, as determined by McSAS, was 3.46 ± 1.16 nm, which is greater than that determined for TEC10V30E alone (see Fig. 3a). This demonstrates that when the catalyst content is low, its apparent size distribution is affected by the presence of carbon mesopores with similar sizes.

Figure 4d shows the scattering curves from the CNovel-TEC10V30E mixture dispersed in 50% TBE. Unlike in the curve from CNovel alone, a Guinier-like feature remained visible, and was analyzable by McSAS. The size distribution was 2.6 ± 0.4 nm, which is close to the value for TEC10V30E alone. This means that by adding a solvent of the matching concentration, the effect of carbon mesopores was eliminated, allowing for a correct evaluation of catalyst particle size distribution.

## Discussion

### Comparison with other techniques

#### Contrast-variation small-angle neutron scattering (CV-SANS)

As explained briefly in Introduction, contrast-variation technique is more widely used in SANS than in SAXS. CV-SANS is also used to study catalysts for fuel cells, but for different purposes^24–27^. In contrast to SAXS, SANS is suitable for discriminating light elements. By utilizing this feature, CV-SANS is used to separate water, ionomer and carbon in the catalyst layer of the fuel cell, by varying the deuterium content in water. CV-SANS can be applied in situ for operating fuel cells. For neutrons, however, the scattering length densities of Pt and carbon are similar, and because of this, CV-SANS cannot be used for determining the particle size distribution of Pt catalysts.

#### Anomalous small-angle X-ray scattering (ASAXS)

This is a method complimentary to CV-SAXS in separating element-specific information in multi-component systems. Both ASAXS and CV-SAXS have merits and demerits, and attention should be paid in considering which method is more suitable for each purpose of measurement.

The largest merit of using ASAXS is that it can be applied to elements heavier than carbon. For example, it can be applied to metal-oxide-supported catalysts^28^. It can also be applied to catalysts with a core–shell or alloy structure, consisting of Pt and cobalt, for example^29^. Another merit is that it can be used for operando measurement of fuel cells. By using this feature, the growth of catalyst particles was monitored during electrochemical testing^30,31^. The demerit of ASAXS is that very strict tuning of X-ray energy is required (5-digit accuracy is necessary). Measurement must be carried out at 3 or more different X-ray energies. For this reason, it is mandatory to use synchrotron-radiation facilities, in which X-ray energies are finely tunable. Reflecting the small amplitude of the anomalous scattering factor, the result is very sensitive to experimental errors, and an error of less than 1% can cause a catastrophic effect^32^. In addition, depending on elements to be measured, the energies at around their absorption edges can be outside the tunable range in particular beamlines.

In contrast, the energy requirement is not very strict for CV-SAXS, although energies just above the absorption edge should be avoided because of increased X-ray absorption and fluorescence. Unlike in ASAXS, multiple energies are not needed. For this reason, CV-SAXS is also suitable for measurements using laboratory X-ray sources.

The demerit of CV-SAXS is that it cannot be applied to heavy elements, and its application is practically limited to carbon-supported metal catalysts. Unlike ASAXS, it cannot be used for operando measurements.

Whether it is CV-SAXS or ASAXS, once the scattering curves for catalysts alone are isolated, they are more suitable for further evaluation of the structural features of catalyst than before isolation. Such analyses include form-factor fitting to shapes other than spheres and determining core–shell structure, surface roughness evaluation, etc. Some of the freely available software packages besides McSAS, such as SasView^33^ and SASfit^34^, have a wide range of form-factor formulae to which experimental data can be fitted.

#### Direct subtraction of support signal

One might think that element-specific signal can be easily obtained by separately recording the scattering from the catalyst-support system and that from the support only, and subtract the latter from the former. If this were a valid method, then there would be no need to do CV-SAXS or ASAXS. Unfortunately, however, this is not a valid method for many reasons. Most importantly, the scattering from the catalyst-support system is not simply a sum of the scattering from the catalyst and that from the support. The catalyst and support are in an interfering distance, and the scattering always contains a cross term (see Supplementary Information for more explanation). Secondly, it is not guaranteed that the carbon structure in the catalyst-carbon system is identical to the carbon sample before conjugation. The processes of conjugation may contain heating above 300 °C, causing a change in the carbon structure such as porosity. Also, removal of catalyst from the carbon surface requires drastic chemical conditions, which will also affect the carbon structure. Thirdly, it is difficult to determine the scaling factor between two powder samples. To determine the scaling factor accurately, the amount of sample in the X-ray beam must be accurately measured, but for powder samples, the density of packing in the cell or capillary is very difficult to control.

#### Transmission electron microscopy (TEM)

TEM is a technique that can directly image individual catalyst particles. The merit of using TEM is, therefore, the shapes of individual particles can be unambiguously determined. Also, aggregated particles are recognized and can be excluded from analysis. To determine the particle size distribution, hundreds to thousands of catalyst particles should be examined for achieving satisfactory statistics. This process is often done manually, and it takes hours to days for analysis. On the other hand, SAXS provides far better statistics, since 100 million catalyst particles exist in the X-ray beam^28^.

### Absence of true matching concentration

In the SAXS measurements of carbon or carbon-supported catalysts, the requirement of monodispersity is evidently not met (polydispersity), because carbon particles of various shapes and sizes are irregularly agglomerated. If these particles have some variations in electron densities, it is expected that they will have different matching concentrations, making it impossible to reach a single matching concentration as a whole. This phenomenon was indeed observed in the present study (as shown by the blue curves in Fig. 2 and Fig. S1). The distribution of electron densities of carbon that explains the experimental data is surprisingly broad (the gray curves in Fig. 2 and Fig. S1). This demonstrates the substantial heterogeneity in the structure of these carbon supports. It is unlikely that this heterogeneity is caused by mesoscopic pores to which the solvent is inaccessible. If such pores are present, features arising from them should be observable in the scattering curve even in the presence of solvent. The heterogeneity was observed for Vulcan, which is solid carbon, and the feature observed for mesoporous CNovel disappeared by addition of the solvent, indicating that its mesopores are accessible for the solvent. If the inaccessible pores are so minute that they fall outside the measured q-range, however, the features arising from them will not be observed, and this possibility cannot be ruled out.

In spite of this heterogeneity, the scattering from the carbon can be reduced to approximately 20% by simply immersing it in DMSO, and it can be further reduced to a few percent at around 50% TBE when compared with the scattering in air. At this concentration, the scattering from carbon is virtually eliminated.

### Choice of contrast agent

As mentioned earlier, the electron density of carbon samples was estimated to be around 1100 mol/L, even higher than that of DNA. This means that the contrast agent that are commonly used in biological SAXS (sucrose and glycerol) cannot be used for the present purposes due to their limited solubility in water. Gabel et al.^35^ suggested the use of highly electron-rich medical contrast agents (iohexol and Gd-HPDO3A) as superior replacements superior for sucrose: they are approximately 2 and 3 times more electron rich, respectively, compared with sucrose. However, even when using Gd-HPDO3A, a concentration of ~ 3 M would be required to match the density of carbon, and again, its limited solubility may prevent its use for the present purposes (refer to their Fig. 1 for its solubility in water).

Unlike biological molecules, the advantage of the carbon-catalyst system is that organic solvents or other water-immiscible solvents can be used for contrast agents. In fact, carbon and catalyst samples disperse more readily in such solvents than in water. Although some stirring effort is required, the present study demonstrates that the carbon/catalyst samples can be uniformly dispersed in the TBE/DMSO mixture, and remain in that state long enough to allow for ordinary SAXS measurements. The highly electron-rich solvent, TBE (1322 mol/L) is found to be the most suitable solvent for the present purposes, although it is unsuitable for biological molecules.

## Conclusion

In this paper, we have developed a method for applying contrast-variation SAXS (CV-SAXS) to evaluate the particle size distribution of metal catalysts on carbon support. We used a mixture of TBE and DMSO as a solvent to match the density of carbon, rendering the carbon invisible to X-rays. Although a strict matching concentration was not achieved, over a wide range of TBE concentrations, the scattering of carbon was reduced to a few percent of that in air. By applying this method to a mesoporous carbon support (CNovel), we demonstrated that the effect of the mesopores can be suppressed, and the particle size distribution of the catalysts can be evaluated without the influence of the mesopores. Considering the simplicity of this method, the CV-SAXS method is expected to be widely employed for characterizing carbon-supported catalyst samples.

### Supplementary Information

Supplementary Information.

## Data Availability

The data used in this study are available upon request to the corresponding author.
