# Supplementary material for: Contrast variation method applied to structural evaluation of catalysts by X-ray small-angle scattering
Source: Sci Rep. 2024 Jan 27;14:2263. doi: 10.1038/s41598-024-52671-7 (PMC10817912; doi:10.1038/s41598-024-52671-7)
Supplement: Supplementary file 1 — Supplementary Information. [file 41598_2024_52671_MOESM1_ESM.pdf]

Supplemental Document 1 for

Contrast variation method applied to structural evaluation of catalysts by X-ray small-angle scattering

Albert Mufundirwa[1], Yoshiharu Sakurai[1], Masazumi Arao[2], Masashi Matsumoto[2], Hideto Imai[2] and Hiroyuki Iwamoto[1*]

[1]Research Project Division, Japan Synchrotron Radiation Research Institute, SPring-8, Sayo-cho, Sayo-gun Hyogo 679-5198, Japan
[2]Fuel Cell Cutting-Edge Research Center Technology Research Association, 3147, Shimomukouyama-cho, Kofu, Yamanashi 400-1507, Japan.

This document contains:

Figure S1. Dependence of the intensity of X-ray scattering from CNovel on the concentration of tetrabromoethane (TBE) in the solvent.
Validation of McSAS with Figure S2.
Comparison with TEM with method description and Figure S3.
Goodness of fit with Figures S4-S9 to show fitted curve by McSAS overlaid on the experimental data, along with magnified histograms.
Cross term between platinum and carbon with Figure S10 to show the effects of the cross term between Pt and carbon on the scattering curves.

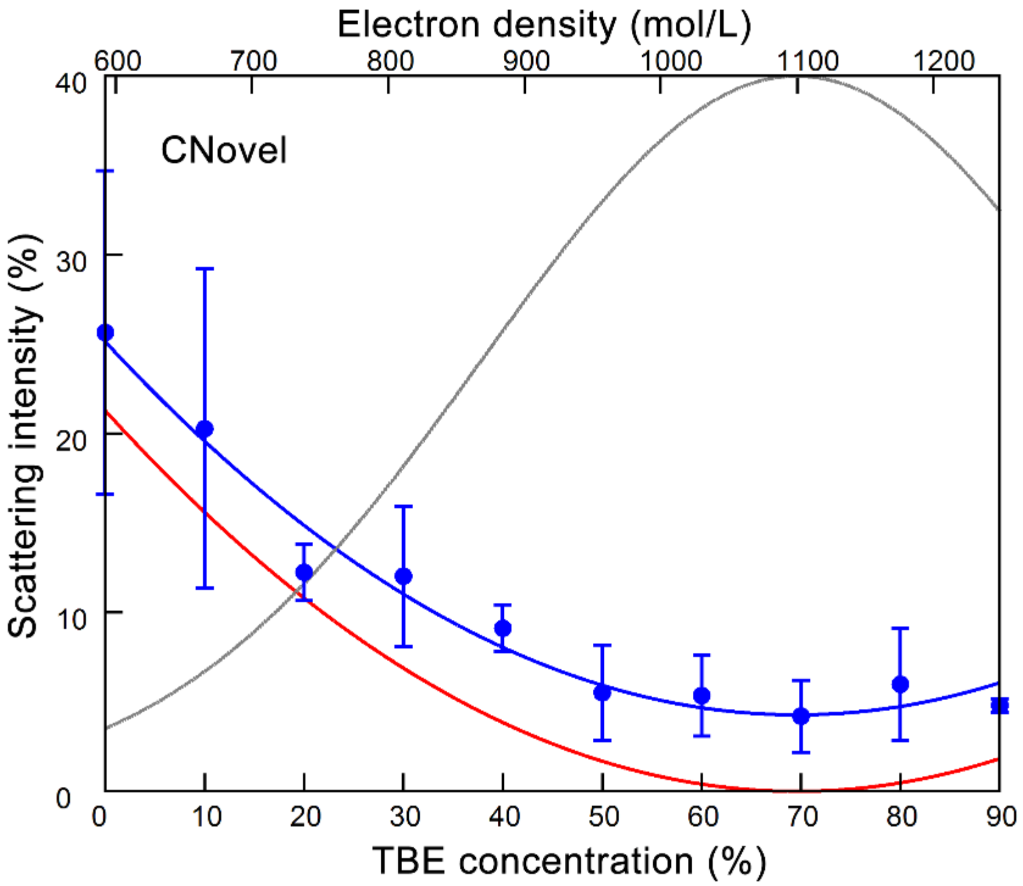

Figure S1. Dependence of the intensity of X-ray scattering from CNovel on the concentration of tetrabromoethane (TBE) in the solvent. n = 3 for all data points. See the legend for text-Fig. 2 for details.

Validation of McSAS

In this study, we used McSAS as a tool for determining the particle size distribution of catalyst samples. Initially, we validated McSAS by using model data with known mean radii and standard deviations. The model in Figure S2 contains spheres with a mean radius of 3 nm and a standard deviation of 0.5 nm (volume fraction). The scattering curve was calculated by using the form factor *F* for a sphere,

$$F = 3\rho V(\sin(qR) - qR\cos(qR))/(qR)^3,$$

where ρ is the density, *V* is the volume of the sphere, *q* is the scattering vector, and *R* is the radius of the sphere.

The calculated scattering curve was subjected to McSAS analysis. The result shows a good agreement between the obtained histogram and the distribution assumed in the model.

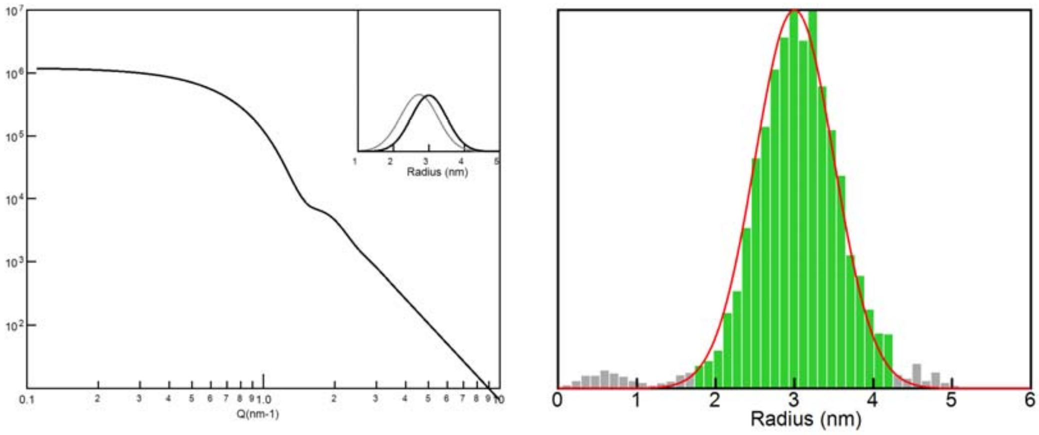

Figure S2. Validation of McSAS by model data. Left, scattering curve calculated from the model containing spheres with radii of 3 ± 0.5 nm. The inset is the assumed particle size distribution (black, volume fraction; gray, particle number). Right, histogram of particle sizes determined by McSAS. The red curve is the distribution originally assumed in the model.

Comparison with TEM

The SAXS results of particle size analysis were compared with the analysis of scanning TEM images. The results of comparison is shown in Fig. S3. For TEM, the available images were from the TEC10V30E samples that had been pretreated with hydrogen gas, so that the SAXS histogram is also from the hydrogen-pretreated sample. The histograms obtained by the two methods show good agreement. However, in SAXS, a small number of larger particles exist. In TEM, it is observed that some particles are aggregated to various extents, and these particles were excluded from analysis. Therefore, the larger particles present in the SAXS histogram are likely to represent these aggregated particles.

Methods: Bright field (BF) and high angle annular dark field (HAADF) STEM images were taken to confirm Pt particle size, using a JEOL JEM-ARM200F transmission electron microscope with accelerating voltage of 200kV. Specimen for TEM observation was prepared by powder dispersion method. Using the obtained STEM images, equivalent circle diameter of Pt particles was measured by image analysis software (Media Cybernetics Image-Pro 10). A total of 454 particles were measured.

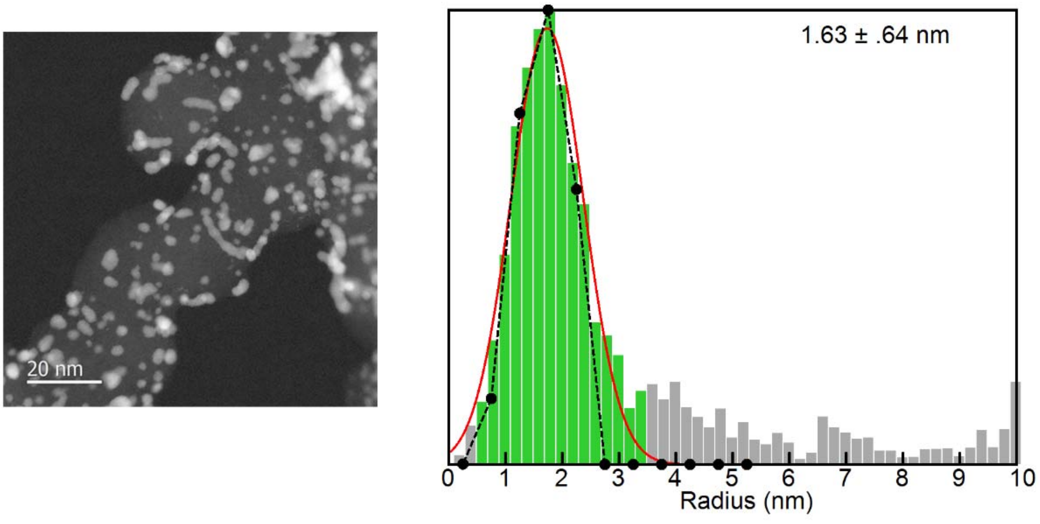

Figure S3. Comparison between TEM and SAXS analyses. Left, a representative dark-field scanning TEM (HAADF-STEM) image of hydrogen-pretreated TEC10V30E; right, result of SAXS analysis (volume fraction) of hydrogen-treated TEC10V30E (green and gray bars) with overlaid TEM volume-fraction histogram (circles and broken lines). The red curve is a Gaussian fit to the SAXS data. The mean radius for TEM is 1.65 nm.

Goodness of fit

The scattering curves from TEC10V30E generally fitted well by McSAS. All the fitted curves coincided well with the measured data from the samples (Figs. S4-S9).

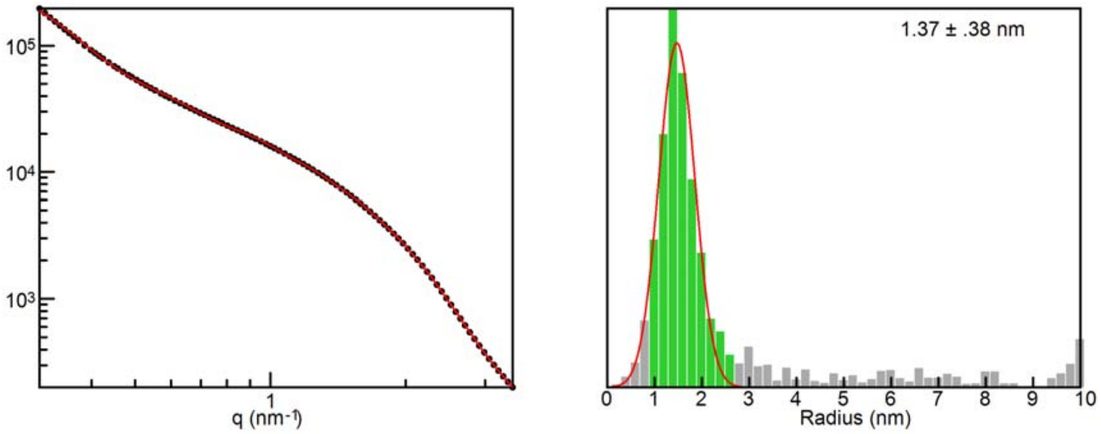

Figure S4. Fitted curve and enlarged particle-size histogram for TEC10V30E powder in the absence of solvent. Left, fitted curve. The output from McSAS was plotted as is. The black dots represent measured intensities. Right, the histogram of particle size distribution obtained by McSAS (Identical to the one in text-Fig. 3a). The red curve is the Gaussian distribution fitted to the histogram.

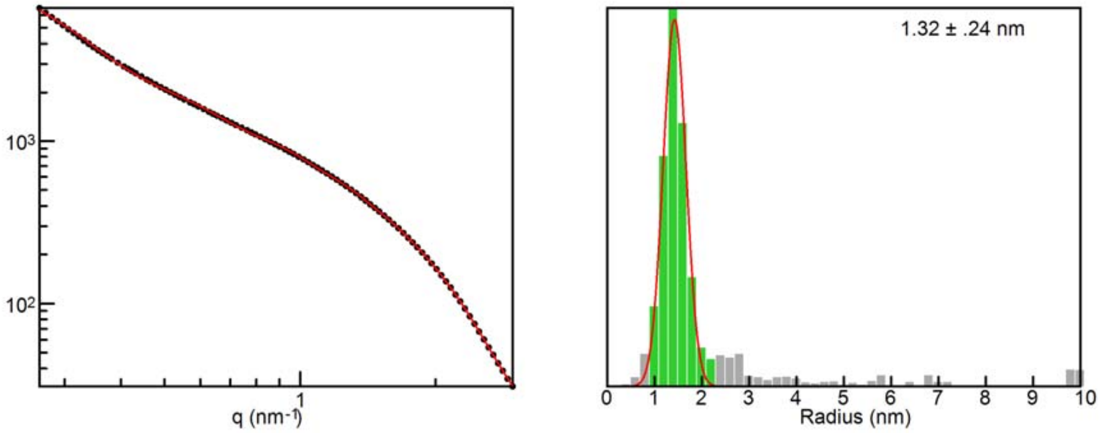

Figure S5. Fitted curve and enlarged particle-size histogram for TEC10V30E in 50% TBE. The histogram is identical to the one in text-Fig. 3b. For details see legend to Fig. S3.

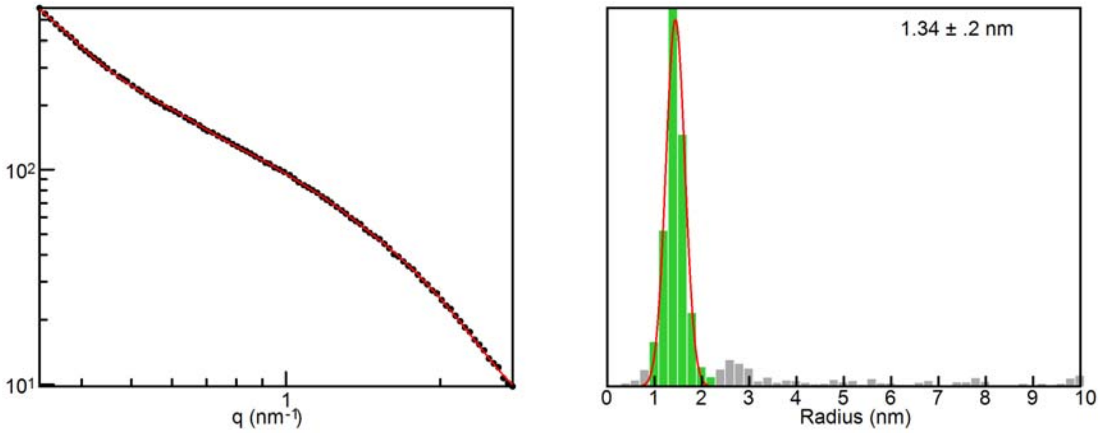

Figure S6. Fitted curve and enlarged particle-size histogram for 5% TEC10V30E and Vulcan in 50% TBE. The histogram is identical to the one in text-Fig. 3d. For details see legend to Fig. S3.

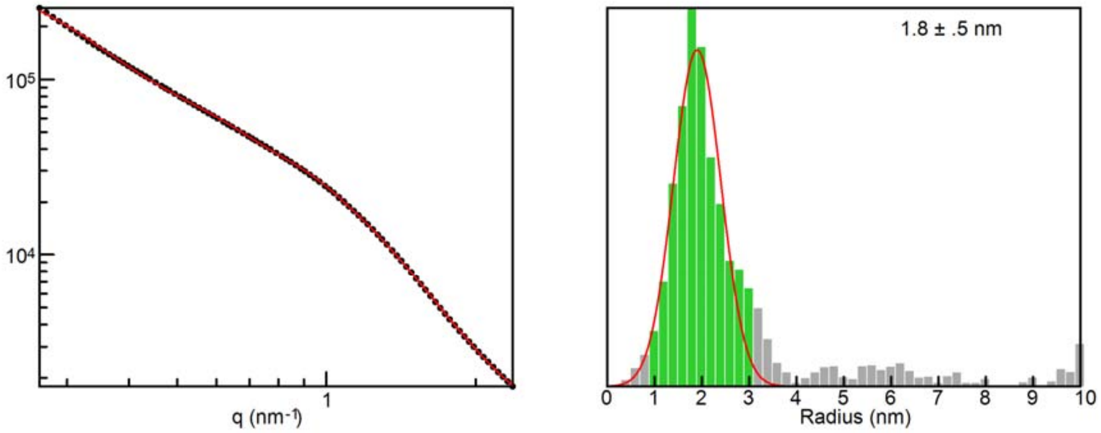

Figure S7. Fitted curve and enlarged particle-size histogram for CNovel powder in the absence of solvent. The histogram is identical to the one in text-Fig. 4a. For details see legend to Fig. S3.

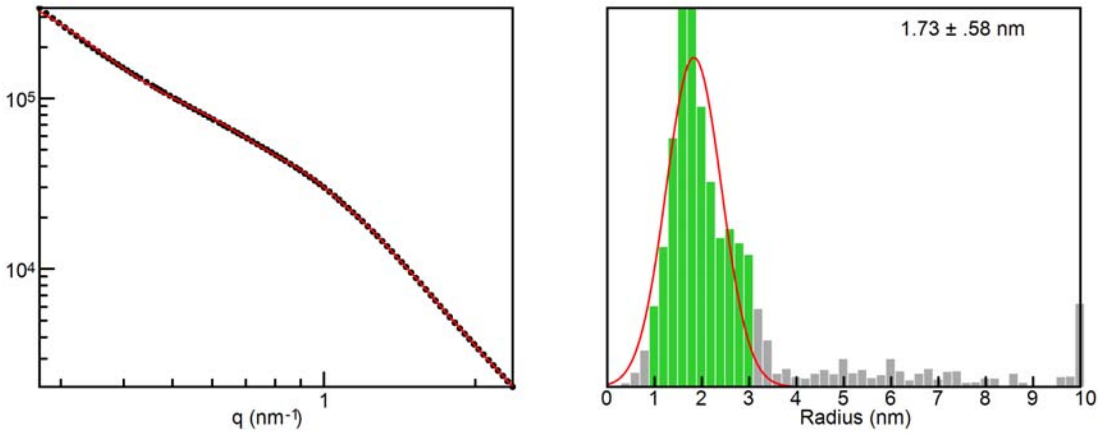

Figure S8. Fitted curve and enlarged particle-size histogram for 15% TEC10V30E and CNovel powder in the absence of solvent. The histogram is identical to the one in text-Fig. 4c. For details see legend to Fig. S3.

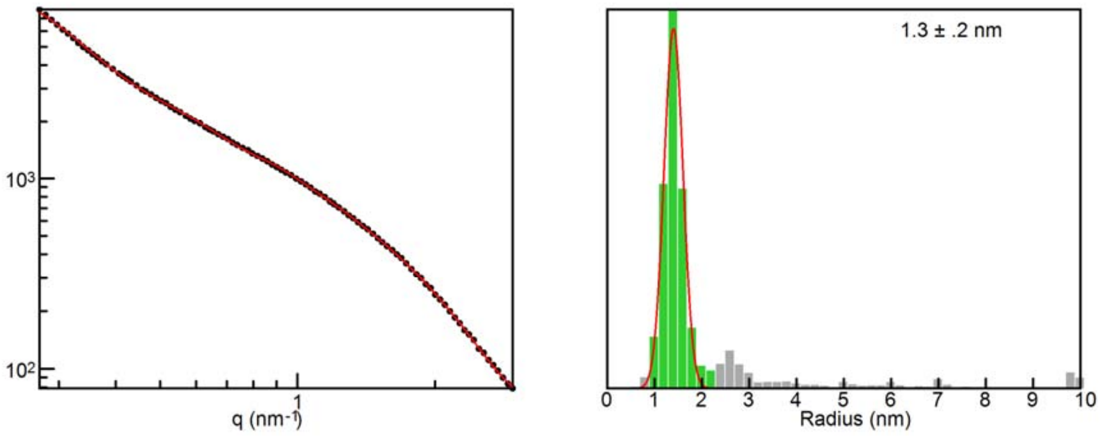

Figure S9. Fitted curve and enlarged particle-size histogram for 15% TEC10V30E and CNovel powder in 50% TBE. The histogram is identical to the one in text-Fig. 4d. For details see legend to Fig. S3.

Cross term between platinum and carbon

To assess the validity of the direct subtraction method, scattering curves were calculated from a model structure. In this model, spherical Pt particles (mean diameter, 3 nm) were randomly placed on the surface of a spherical solid carbon support (diameter, 30 nm). The scattering curves were calculated for 4 different cases.

(1) Scattering from the full structure of Pt particles on the carbon support (Fig. S10, blue curve). This is the curve obtained from usual recordings of carbon-supported catalyst samples filled in capillaries, etc.

(2) Scattering from the Pt particles alone (Fig. S10, green curve). Except for the absence of the carbon support, the sizes and distribution of the Pt particles are identical to those in case (1). This is the scattering curve obtained from carbon-supported catalyst samples in a matching solvent.

(3) Scattering from the carbon support alone, without Pt particles (Fig. S10, black curve).

(4) Scattering from the full structure of Pt particles on the carbon support, from which the scattering from the carbon support (3) has been subtracted (Fig. S10, red curve). This is the curve obtained by the direct subtraction method.

If the direct subtraction method were a valid method, the curve obtained by this method (4) should coincide with the true curve (2). In reality, however, a large error exists, especially in the low-q region. At the lowest q-value in this calculation, the scattering intensity of (4) is ca. 2.4 times greater than the correct value (2). This indicates that, in the q-range where there are substantial contributions from carbon, the cross term between Pt and carbon can never be ignored. If it is mesoporous carbon, it is expected that the cross term will remain high in the higher-q region.

In addition, it is noticed that the slope at the low-q end of the Pt-alone curve (Fig. S10, green) is not flat, contrary to what is expected from the form factor of a sphere. This is because the low-q part of the curve reflects the higher-order structure of the carbon-supported catalyst particles, which takes on a core-shell-like form. In most samples, the diameter of carbon particles is far greater than that of Pt particles, and this higher-order structure will have little effect on the estimated Pt particle size distribution.

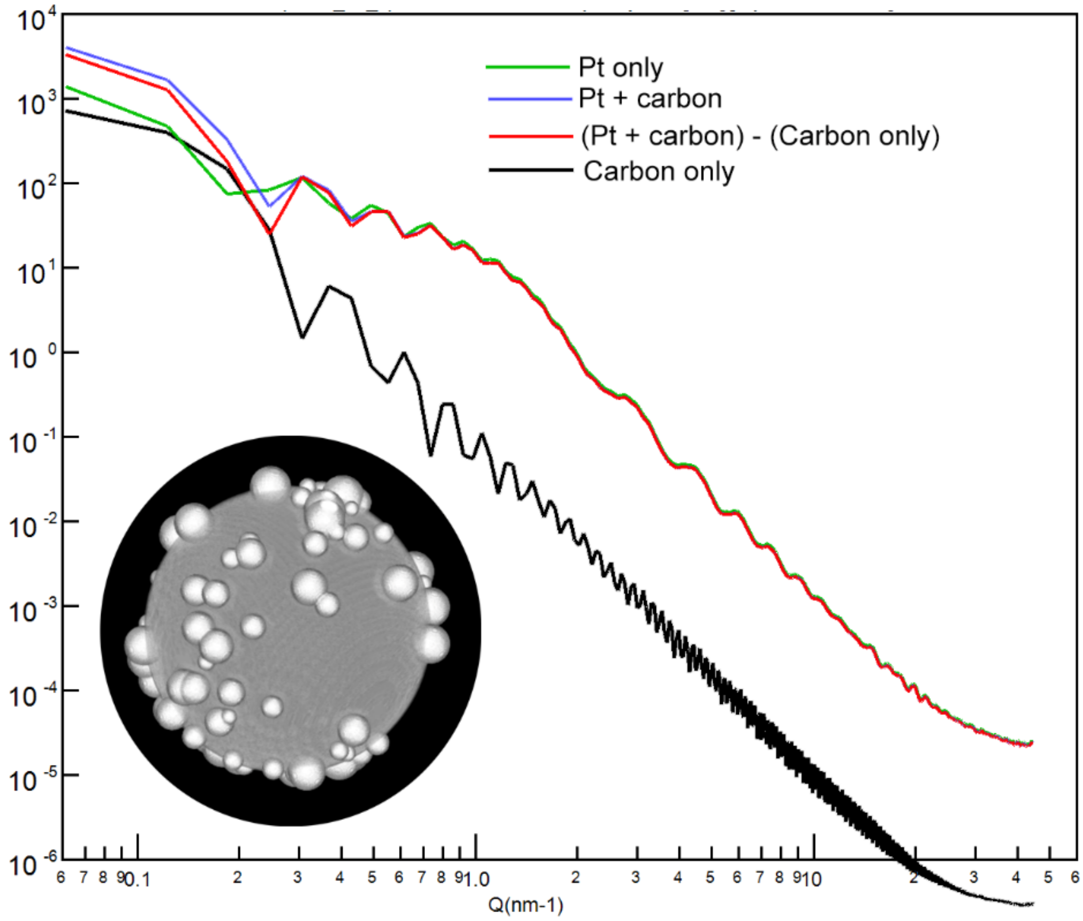

Figure S10. Scattering curves calculated from a model structure of Pt particles supported on the surface of a solid carbon sphere. The diameter of the carbon is 30 nm, and the average diameter of the 100 Pt particles is 3 nm. Green, scattering from Pt alone; blue, scattering of the whole structure (Pt on carbon); red, subtraction of carbon-only curve from the Pt-on-carbon curve; black, scattering from carbon alone. Inset is a 3D-rendered view of the model structure. Note a substantial discrepancy between the Pt-only curve (green; correct curve) and the curve obtained by direct subtraction (red), especially at the low-q region. The model structure was placed in a 1024 x 1024 x 1024 voxel space, and the scattering curves were calculated by performing 3D fast Fourier transformation.
